# A comparative study of the clinical efficacy of supination-adduction type II ankle fracture surgery based on the medial pilon fracture concept versus the ankle fracture concept

**DOI:** 10.1186/s12891-021-04818-0

**Published:** 2021-11-10

**Authors:** Zhida Ma, Junfeng Zhan, Nan Zhu, Liujie Zheng, Yao Hu, Wei Liu, Jinxin Li, Juehua Jing

**Affiliations:** Department of Orthopaedic Surgery, The Second Hospital of Anhui Medical University, No. 678 Furong Road, Economic and Technological Development Zone, Hefei, 230601 China

**Keywords:** Ankle fracture, Supination-adduction (SAD) fracture, Medial pilon fracture

## Abstract

**Background:**

A supination-adduction (SAD) ankle fracture is a special type of ankle fracture that results in collapse of the distal tibial articular surface; as such, orthopaedic surgeons require greater awareness of this type of fracture. The severity of this injury lies between that of an ordinary ankle fracture and a pilon fracture, and the treatment of such fractures based on the ankle fracture concept leads to extremely high rates of postoperative complications and a poor prognosis. In this retrospective study, we aimed to explore the treatment of SAD fractures based on the pilon fracture concept.

**Methods:**

We retrospectively analysed the clinical data of 67 patients with Lauge-Hansen supination-adduction type II (SAD-II) ankle fractures, most of whom had a 44-A AO classification. Patients underwent surgical treatment at the Second Affiliated Hospital of Anhui Medical University from January 2009 to June 2019. The patients were divided into two groups based on the surgical concept employed: 43 patients were included in the ankle fracture surgical concept group, and 24 patients were included in the medial pilon fracture surgical concept group. The therapeutic effect was evaluated based on the Burwell-Charnley radiological reduction standard, the American Orthopaedic Foot and Ankle Society (AOFAS) ankle-hindfoot score and postoperative visual analogue scale (VAS) pain score 1 year after surgery using regression with adjustment for confounding factors.

**Results:**

All 67 patients were followed up. Twenty-four patients were treated according to the medial pilon fracture concept, and forty-three patients were treated according to the ankle fracture concept. The AOFAS score 1 year after surgery in the medial pilon group (89.83 ± 2.77) was higher than that in the ankle fracture group (83.63 ± 7.97) (*p* < 0.05). The VAS score 1 year after surgery in the medial pilon fracture group (1.17 ± 0.96) was significantly better than that in the ankle fracture group (2.28 ± 0.96) (*p* < 0.05).

**Conclusion:**

Patients with Lauge-Hansen SAD-II ankle fractures treated based on the medial pilon fracture surgical concept had better postoperative outcomes than those treated based on the ankle fracture surgical concept.

**Level of evidence:**

Level III, retrospective cohort study.

## Introduction

Ankle fractures are common fractures that result from lower limb trauma, accounting for 7% of all fractures [1, 2]. Ankle fractures are low-energy injuries associated with rotational forces. According to the Lauge-Hansen classification system, supination-adduction (SAD) [3] fractures are rarely reported, accounting for only 10–20% of ankle fractures [4]. Of SAD fractures, supination-adduction type II (SAD-II) fractures are characterized by loss of ankle stability. The talus impacts the medial plate of the tibia to cause oblique, oblique inward upward or vertical upward fractures combined with bone compression, collapse, or cartilage surface damage to the ankle vault due to serious damage to the ankle [5]. If the injury is not accurately diagnosed and treated with open anatomical reduction and internal fixation, loss of joint integrity may occur, resulting in severe traumatic sequelae, permanent ankle pain and greatly reduced quality of life [6, 7].

**Fig. 1:**
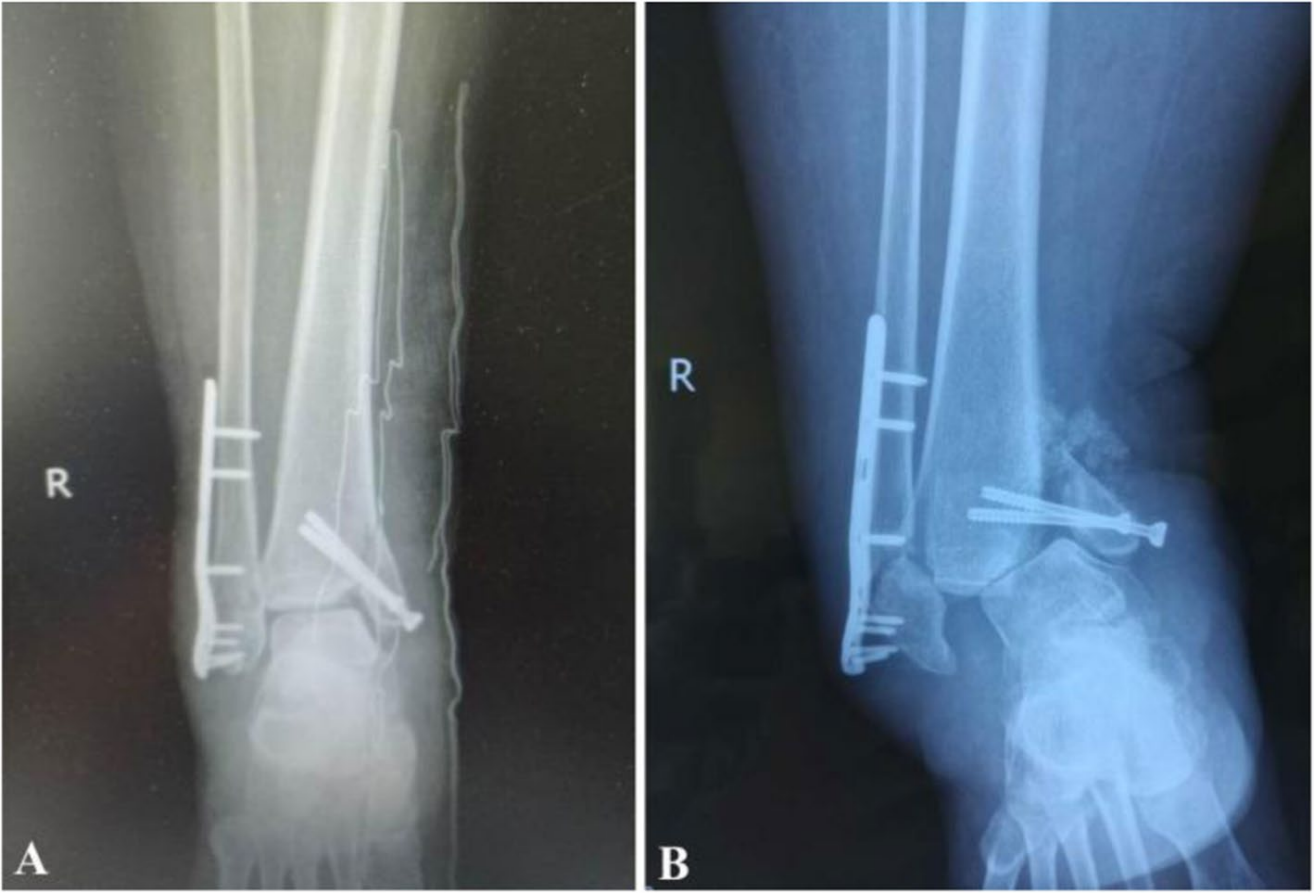
A typical case of internal fixation failure. A. Postoperative radiographs showing that the medial malleolus was only fixed with hollow screws, with no anti-skid plates or screws used for fixation. The collapse of the medial malleolus was not adequately treated with bone grafting. B. Two months after the operation, the internal fixation was found to have failed after a sprain while walking

The peculiar shape of the medial malleolus leads to a high failure rate for internal fixation (Fig. 1), redisplacement of the fracture, varus deformity of the ankle, and postoperative traumatic arthritis [8]. Thus, we hypothesized that the surgical concept used for ankle fractures may not be the best for the surgical treatment of this kind of fracture; rather, the uniqueness SAD ankle fractures may make them more similar to pilon fractures [9]. Unlike common ankle fractures, pilon fractures are very complex and involve the articular surface and tibial shaft. These fractures are characterized by compression fractures and articular comminution of the distal metaphysis [10]. The treatment of SAD ankle fractures with distal tibial collapse should follow the basic principles of pilon fracture treatment [11], emphasizing the importance of reduction and anti-skid plate or anti-skid screw implementation to improve ankle fractures with medial collapse. Referring to Hansen et al’s definition of posterior malleolar fractures on the coronal plane as posterior pilon fractures in 2000 [12], we describe fractures between medial ankle joint fractures and pilon fractures as “medial pilon fractures.” Therefore, one should consider whether to follow the surgical concept used for ankle fractures or for medial pilon fractures.

One purpose of this study was to evaluate the prognosis of patients with SAD-II ankle fractures treated based on the ankle fracture concept compared with the pilon fracture concept. To do so, we compared the results of imaging and clinical evaluations. The other purpose of the present study was to identify SAD-II ankle fractures based on plain radiographs and to explore fracture characteristics to guide surgical treatment selection.

## Methods

The institutional review committee approved the study prior to its initiation. Informed consent was obtained from all participants included in the study. We retrospectively analysed radiographs from 67 adult patients with SAD-II ankle fractures. The patients were selected by two ankle surgeons after screening from January 2009 to June 2019. The AO classification was also used in these cases [13]. The inclusion criteria were as follows: (1) SAD-II ankle fracture based on the Lange Hanson classification; (2) age ≥ 18 years and ≤ 60 years; and (3) fresh closed fracture. The exclusion criteria were as follows: (1) a history of ankle or talus fracture, which may affect postoperative ankle function; (2) open fracture; and (3) advanced age or a serious underlying disease rendering the patient unable to tolerate surgery. Finally, 43 patients (ankle fracture group) were treated based on the ankle fracture concept, including 11 44-A1 patients, 17 44-A2 patients, 9 44-B1 patients, and 6 44-B2 patients. The remaining 24 patients (medial pilon fracture group) were treated based on the medial pilon surgical concept and included 8 44-A1, 8 44-A2, 6 44-B1, and 2 44-B2 patients. The minimum follow-up time for each patient was 1 year.

### Operative techniques

#### Preoperative management

Before the operation, all affected limbs were examined by plain anteroposterior and lateral radiography and computed tomography (CT) with coronal, sagittal and articular surface reconstruction. The time from injury to operation was recorded in the two groups. All operations were performed after the doctor identified detumescence of the affected limb.

#### Operative management

**Fig. 2:**
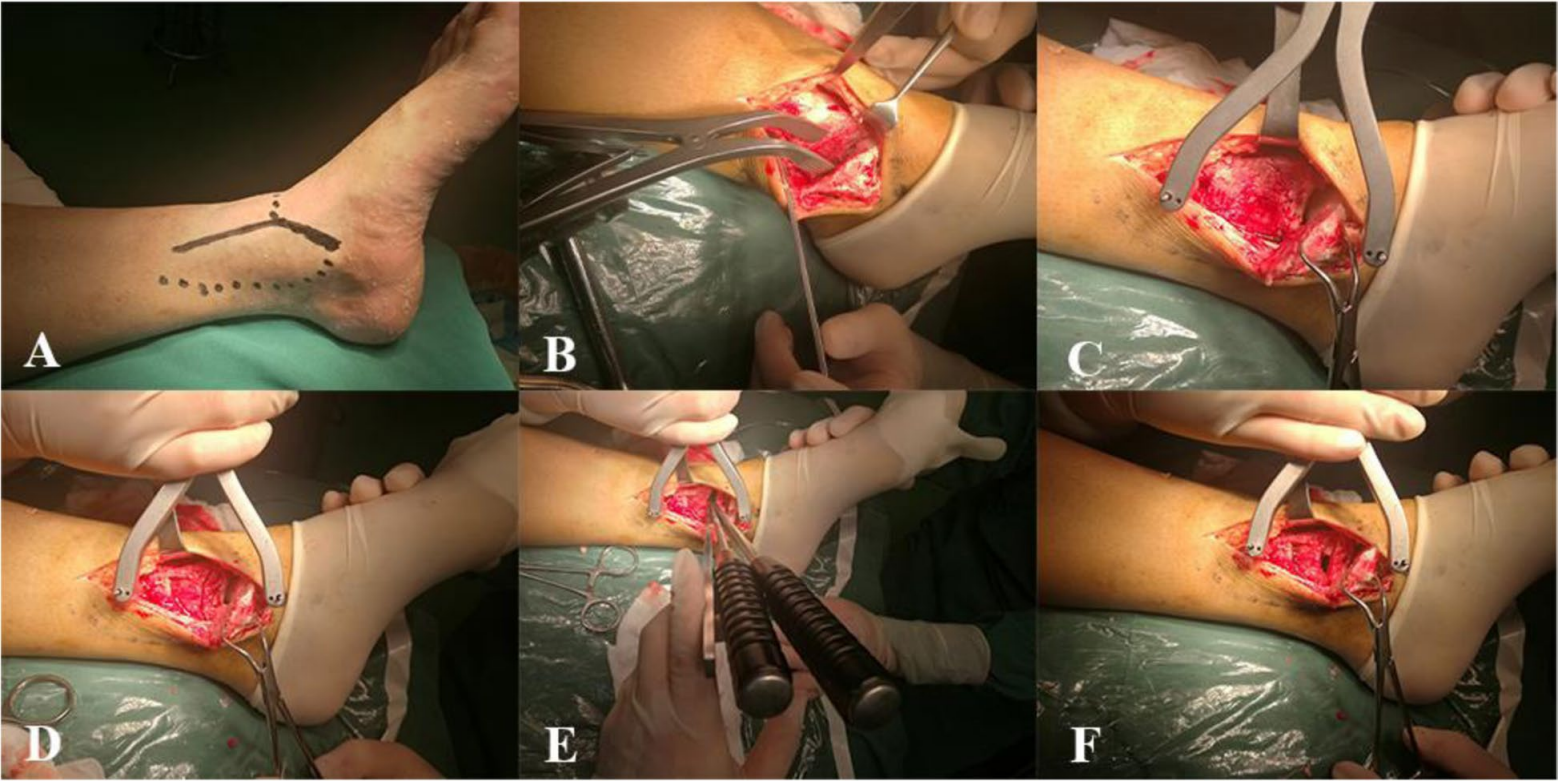
In the medial pilon fracture group,open the joint capsule, the distal collapse of the tibial bone was visible during the operation, restoring articular surface leveling and adequate bone grafting

With the patient in the supine position under general anaesthesia, a pressurized tourniquet was placed at the thigh root of the affected limb, and a tincture of iodine was used for routine disinfection followed by draping. Then, the tourniquet was pressurized to 260 mmHg. An arc incision was made in the anterolateral malleolus of the affected limb to expose the transverse fracture end of the lateral malleolus. After removal of the soft tissue and coagulated blood at the fracture end and reduction under direct vision, a distal fibular anatomical plate was used for fixation. Then, an arc-shaped incision was made on the posteromedial side of the medial malleolus to expose the medial malleolus. In the ankle fracture group, part of the medial malleolus was exposed to clean and reset the anteromedial collapsed articular surface without opening the joint capsule. An allogeneic cancellous bone graft was applied to the collapsed area, and two K-wires were used for temporary fixation, and a 1/3 tubular steel plate or a cannulated screw was used to fix the medial malleolus. In the medial pilon fracture group (Fig. 2), the medial malleolus was exposed, and the joint capsule was opened. Two K-wires were then inserted into the talus and tibia, and a distraction device was used to open the medial malleolus. The medial articular surface of the tibia was obviously collapsed when the medial malleolus was opened. Three osteotomes were used to stack the collapsed fracture until the articular surface was leveled. Temporary transdermal fixation was performed with 1.2 or 1.5 K-wires,then allogeneic cancellous bone was implanted to completely fill the cavity under the articular surface, providing sufficient mechanical support. A 1/3 tubular steel plate was fixed on the medial side to prevent sliding, and a cannulated screw was inserted into the guide wire to fix the medial malleolus. The operative field was washed after fixation suitability was verified by fluoroscopy, and the incision was closed layer by layer. Finally, a short plaster leg support was applied for patients in the medial pilon fracture group.

#### Postoperative management

The wound dressing was changed 24 h after the operation, and measures for analgesia and detumescence were administered for 3 days. The sutures were removed 2–3 weeks after the operation. In the ankle fracture group, the patients gradually began to walk with full weight-bearing after 4 weeks. And in the medial pilon fracture group, the patients gradually increased weight-bearing under supervision of the surgeon after 4 weeks, with full weight-bearing after 8 weeks.

### Follow-up evaluation

At the postoperative follow-up, wound healing was evaluated, and plain anteroposterior and lateral ankle radiographs were reviewed to observe the fracture fragment positioning and healing at 4 weeks, 8 weeks, 6 months and 12 months postoperatively. The reduction condition was evaluated according to the Burwell-Charnley radiological reduction criteria, and the rate of excellent and good outcomes was determined [14]. The visual analogue scale (VAS) pain score and American Orthopaedic foot and Ankle Society (AOFAS) ankle joint-hindfoot function score were determined 1 year after surgery. All evaluations were performed by two or more attending foot and ankle surgeons.

### Statistical methods

SPSS software (version 26.0) was used to input data. Chi-square tests and t-tests were used for between-group comparisons. Factors with significant differences between groups (*P* < 0.05) and *P* < 0.2 in the univariate linear regression analysis were included in the multiple linear regression model to adjust for confounding factors and clarify the influence of the surgical concept on the AOFAS and VAS scores. Variables were added to the model using the enter method. Differences were considered statistically significant at *P* < 0.05.

## Results

All the 67 patients were followed up for an average of 14 months (range, 12–16 months). There were 43 patients in the ankle fracture group and 24 patients in the medial pilon group. More detal information is shown in Table 1.

**Table 1:** Differences in the distribution of ankle fracture group VS medial pilon fractures group among different populations

Characteristics	Ankle Fracture Group	Medial Pilon Fractures Group	***P***-Value
	***n*** = 43	***n*** = 24	
Gender (female/male)	13/30	20/4	< 0.001*
Age (yr)	33.42 ± 12.85	33.88 ± 14.64	0.895
Position (left/right)	11/32	9/15	0.307
Smoker (n, [%])	10(20.9%)	14(58.3%)	0.004*
Diabetes (n, [%])	2(4.7%)	2(8.3%)	0.542
Time from injury to surgery (hours)	5.67 ± 1.15	5.92 ± 1.35	0.462
BMI (kg/m2)	25.82 ± 1.68	25.78 ± 2.40	0.948
Postoperative complications (n, [%])	18(41.9%)	3(12.5%)	0.013*
Burwell-Charnley Radiological Reduction Standard (excellent/good/poor)	38/5	23/1	0.562
AO-classification (A1/A2/B1/B2)	11/17/9/6	8/8/6/2	0.800
AOFAS score one year after surgery(points)	83.63 ± 7.97	89.83 ± 2.77	< 0.001
VAS score one year after surgery(points)	2.28 ± 0.96	1.17 ± 0.96	< 0.001

Categorical variables are expressed as (n / %), Quantitative variables are expressed as mean ± SD.BMI indicates body mass index.

It demonstrated that gender and operation mode had significant influence on the AOFAS score 1 year after surgery through univariate linear regression analysis by exploring the influence of surgical methods on the AOFAS score 1 year after surgery (Table 2). Multivariate regression analysis showed that the operation mode affects the AOFAS score 1 year after surgery independently (Table 3). At the same time, in the analysis of the impact of surgical concept on the VAS score 1 year after surgery, it showed that gender and surgical concept had significant differences on the VAS score 1 year after surgery by univariate linear regression analysis (Table 4). After adjusting sex factor, we found that there were significant differences in the effect of different surgical methods on the VAS score 1 year after surgery (Table 5).

**Table 2:** Univariate linear regression results with the AOFAS score one year after surgery before and after surgery as the dependent variable

Characteristics	Partial regression coefficient	Standard error	Standard partial regression coefficient	T value	***P*** value
Gender^a^	−4.355	1.694	−0.304	−2.570	< 0.012
Age	0.063	0.066	0.118	0.955	0.343
Position^a^	−1.211	1.937	−0.077	−0.625	0.534
Smoker	0.752	1.852	0.050	0.406	0.686
Diabetes	1.488	3.748	0.049	0.397	0.693
Time from injury to surgery	0.994	0.724	0.168	1.373	0.174
BMI	0.374	0.458	0.101	0.817	0.417
Surgical concept^a^	6.205	1.687	0.415	3.678	< 0.001

^a^The gender is male as the control, the orientation is the left side as the control, the operation method is the ankle joint group as the control

**Table 3:** Multivariate linear regression results with the AOFAS score one year after surgery as the dependent variable

Characteristics	Partial regression coefficient	Standard error	Standard partial regression coefficient	T value	***P*** value
Constant	81.406	4.847	–	16.795	< 0.001
Gender^a^	−1.787	1.881	−0.125	−0.950	0.346
Surgical concept^a^	5.256	1.962	0.352	2.679	0.009

^a^The gender is male as the control, the orientation is the left side as the control, the operation method is the ankle joint group as the control

**Table 4:** Univariate linear regression results with the VAS score one year after surgery as the dependent variable

Characteristics	Partial regression coefficient	Standard error	Standard partial regression coefficient	T value	***P*** value
Gender^a^	0.720	0.254	0.331	2.833	0.006
Age	−0.007	0.010	− 0.084	− 0.680	0.499
Position^a^	0.115	0.294	0.048	0.391	0.697
Smoker	−0.528	0.273	−0.233	−1.933	0.058
Diabetes	−0.671	0.562	−0.146	−1.192	0.237
Time from injury to surgery	−0.091	0.111	−0.101	−0.820	0.415
BMI	0.002	0.070	0.003	0.025	0.980
Surgical concept^a^	−1.112	0.245	−0.491	−4.545	< 0.001

^a^The gender is male as the control, the orientation is the left side as the control, the operation method is the ankle joint group as the control

**Table 5:** Multivariate linear regression results with the VAS score one year after surgery as the dependent variable

Characteristics	Partial regression coefficient	Standard error	Standard partial regression coefficient	T value	***P*** value
Constant	2.919	0.732	–	3.986	< 0.001
Gender^a^	0.207	0.292	0.095	0.709	0.481
Smoker	−0.091	0.280	−0.040	−0.326	0.745
Surgical concept^a^	−0.970	0.291	−0.428	−3.339	0.001

^a^The gender is male as the control, the orientation is the left side as the control, the operation method is the ankle joint group as the control

## Discussion

**Fig. 3:**
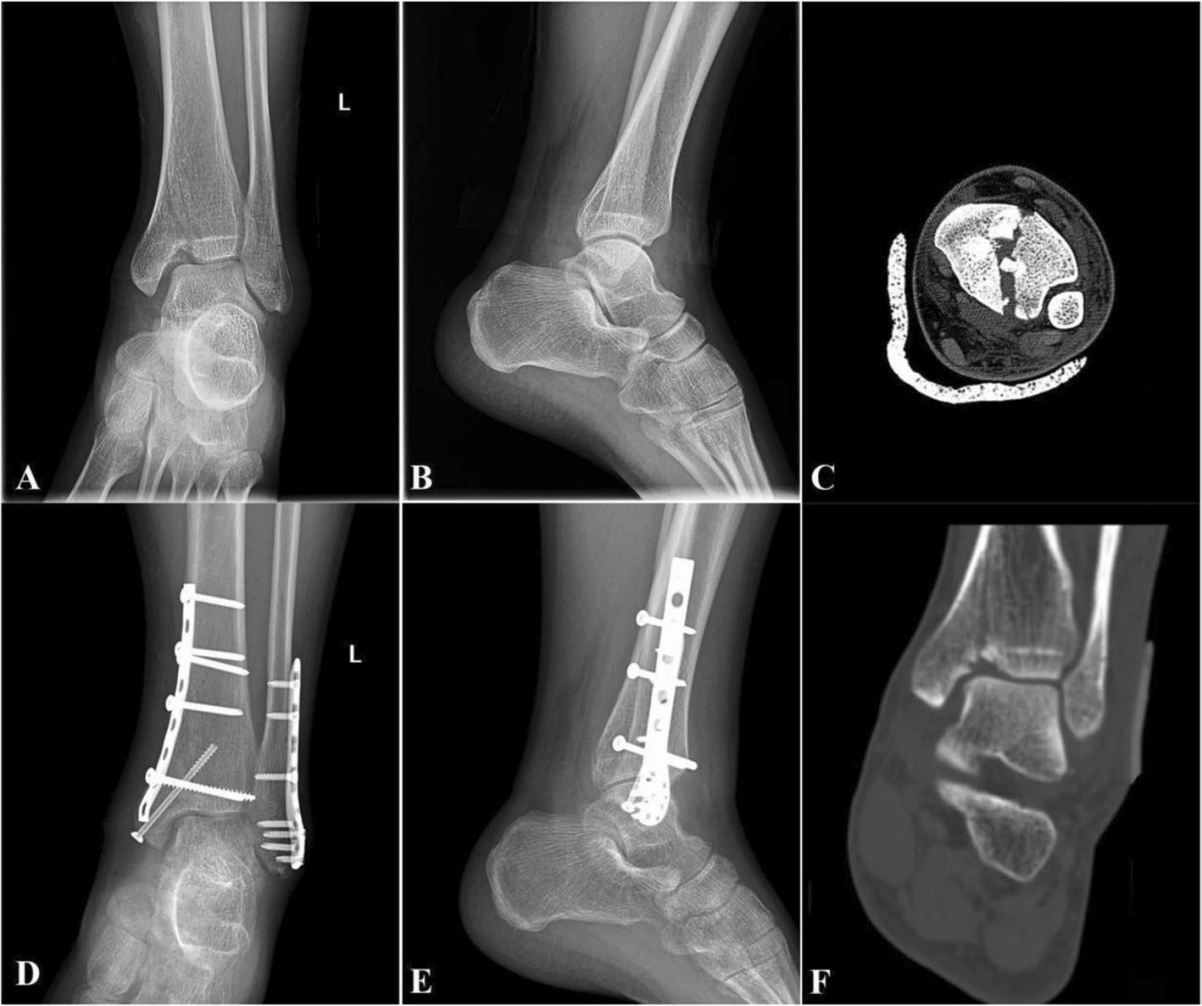
A and B show the “horizontal” fracture line of the lateral malleolus and the “vertical” fracture line of the medial malleolus and the collapse of the articular surface of the distal tibia. C shows the die-punch bone block in the axial CT scan. D and E are the anterior and lateral radiographs of the medial pilon fracture group after surgery, and the ankle joint is stabilized. F shows the sagittal plane CT scan confirming the vault collapse of the articular surface of distal tibia shown in the X-ray plain film

SAD ankle fractures are a very rare type of fracture, and SAD-II fractures are even rarer. Due to its anatomy, varus movement of the talus due to rotation and vertical forces results in fracture and collapse of the articular surface of the distal tibia to form a vertical fracture line during the injury. This special type of fracture has characteristics that other traditional ankle fractures do not have, as follows: 1) lateral collateral ligament injury; 2) subchondral bone and cartilage injury of the medial malleolus; 3) a horizontal lateral fracture line of the ankle and vertical fracture line of the medial malleolus; 4) a die-punch-like fragment of the medial malleolus evident on CT; and 5) injury to the articular surface of the distal tibia and vault collapse (Fig. 3).These features are not common in traditional rotational ankle fractures, but their important contribution to collapse is often ignored in clinical practice [6, 7, 15]. There is no accurate name for a type of fracture that involves the impaction of a medial triangular fragment on the lateral articular surface of the tibia. In our practice, we have described such fractures as medial pilon fractures, which are characterized by transverse fracture of the lateral malleolus and vertical fracture of the medial tibia. Consequently, the articular surface is obviously depressed at the proximal end, as observed on X-ray examination. X-rays of ankle fractures have shown high predictive value for bone and ligamentous injuries [16]. Increasing our understanding of SAD fractures, especially SAD-II fractures involving collapse of the articular surface, may change the surgical concept and bone graft that ankle surgeons choose to use.

Recently, concerns about SAD ankle fractures have increased. Due to the medial vertical fractures caused by the unique injury mechanism and the high rate of postoperative complications associated with this fracture, treatment by open reduction and internal fixation has been accepted by most scholars [8, 17]. However, there is almost no consensus on the indications for a medial malleolar operation or the reduction or fixation method used. Wegner and Ebraheim showed that the mechanical strength of anti-sliding plate fixation for the treatment of a vertical medial malleolar fracture was significantly better than that of monocortical or bicortical fixation [18, 19]. Jones et al. found that the addition of a screw to the vertical fracture line at the distal end of the anti-slide plate significantly enhanced the shear strength [20]. Although distal tibial buttress plate fixation or screw vertical medial malleolar fracture line fixation are the accepted surgical treatments for these fractures, the existence of a die-punch-like bone fragment of the medial malleolus should be an important consideration when stabilizing the ankle joint to restore joint integrity by surgical intervention. The high rates of postoperative complications and poor functional recovery after surgical approaches based on the ankle fracture concept should suggest consideration of the pilon fracture concept as a superior surgical approach for the treatment of these ankle fractures. In 1979, Rüedi and Allgöwer proposed four basic principles of open reduction and internal fixation for pilon fractures, namely, reconstruction and restoration of the fibular length, reconstruction of the articular surface, metaphyseal bone grafting and internal fixation [21]. The treatment of such fractures based on the medial pilon concept focuses on direct reduction (including reduction of the die-punch-like bone fragment), bone grafting and strong internal fixation. It requires anatomical reduction of the articular surface and restores the force line and length of the lower limb, which helps to restore ankle joint stability.

After X-ray examination, SAD-II ankle fractures can be diagnosed by CT. Therefore, CT should be performed to make a clear diagnosis prior to surgery. Evaluating the ankle after fracture using imaging can better inform the selection of the surgical concept. SAD-II ankle fractures require anatomical reduction, proper fixation and articular surface repair for a successful outcome. The degree of articular surface collapse and distal tibial cartilage injury should be carefully evaluated during the operation. The joint capsule should be opened, examined for cartilage fragments and cleaned thoroughly to reduce the risk of postoperative complications. Protection of the soft tissue during surgery is also very important to provide strong support for the collapsed cartilage surface and prevent later collapse of the cartilage.

The analysis of two treatment concepts for Lauge-Hansen SAD-II ankle fractures showed that the postoperative AOFAS and VAS scores were significantly different between the two groups, with better function of the affected limb after surgery in the medial pilon group. This study offers significant guidance for the clinical diagnosis of this kind of fracture, the selection of the treatment method, the main operative points, the postoperative rehabilitation exercise regimen and the prevention and treatment of complications. Therefore, understanding SAD-II fractures and selecting the treatment plan based on the appropriate treatment concept has high clinical significance.

Notably, there are some limitations to this study. These include the inherent weaknesses of a retrospective study. The small sample size limits the generalizability of the results. Future studies with a larger patient population, including a multicentre investigation, are needed to confirm the results of the present study and demonstrate repeatability and generalizability.

In conclusion, patients with Lauge-Hansen SAD-II ankle fractures treated based on the medial pilon fracture surgical concept had better postoperative outcomes than those treated based on the ankle fracture surgical concept.

## Data Availability

The datasets used and/or analysed during the current study are available from the corresponding author on reasonable request.

## Abbreviations

AOFAS: American orthopaedic foot and ankle society
SAD-II: Supination-adduction type II
VAS: Visual analogue scale
